# Relationship quality among dating adolescents: Development and validation of the Relationship Quality Inventory for Adolescents

**DOI:** 10.3389/fpsyg.2022.1026507

**Published:** 2022-10-14

**Authors:** Andréanne Fortin, Laurie Fortin, Alison Paradis, Martine Hébert

**Affiliations:** ^1^Département de Psychologie, Université du Québec à Montréal, Montréal, QC, Canada; ^2^Département de Sexologie, Université du Québec à Montréal, Montréal, QC, Canada

**Keywords:** relationship quality, dating relationships, adolescence, instrument development, validation, psychometric properties

## Abstract

Relationship quality has implications for individuals’ and couples’ wellbeing, such as higher couple functioning and perceived quality of life. In adolescence, low relationship quality has been associated with poor mental health and relational outcomes. However, given the lack of instruments to assess satisfaction in dating relationships, most studies have relied on measures of marital satisfaction. The current study aimed to address this gap by elaborating and validating the Relationship Quality Inventory for Adolescents (RQI-A). Exploratory and confirmatory factor analyses were conducted among two samples of French-speaking dating adolescents (*n*_1_ = 310; *n*_2_ = 335). The two-factor structure (Connectedness and Commitment) was cross-validated, and dimensions showed high internal consistency coefficients (ω = 0.86–0.89). Results also provide evidence of convergent validity of the scale with related measures. The RQI-A may help study predictors and correlates of dating relationship quality.

## Introduction

Dating relationships play a pivotal role in adolescent socioemotional development by offering them working models of intimate relationships, which also serve as a foundation for future relationships (Furman, 2018). Given its importance for psychological adjustment and the stability and durability of romantic relationships, relationship quality is central to studying individual and couple wellbeing (Breitenstein et al., 2018; Pieh et al., 2020). Nevertheless, correlates of dating relationship quality remain understudied as previous research has mainly focused on the associated outcomes of romantic involvement (Davila, 2008). Available findings among adolescent samples have shown that low dating relationship quality is associated with attachment insecurities, difficulties in managing conflicts, and dating violence (DV) (Mikulincer and Shaver, 2013; Orpinas et al., 2013; Todorov et al., 2021). On the other hand, high dating relationship quality has been linked with better problem-solving and relational skills (Furman et al., 2008; Todorov et al., 2021). However, these studies have relied on measures of marital relationship quality, which have not been validated among dating adolescent samples; hence results may be biased by developmental differences between these two types of relationships.

Since dating and marital relationships differ in numerous aspects (e.g., household chores, childcare, or financial issues), indicators of relationship quality may also differ between adolescence and adulthood. Satisfaction and commitment are standard measures of relationship quality (Morry and Sucharyna, 2019). *Satisfaction* can be defined as one’s subjective evaluation of the global relationship (Keizer, 2014), whereas, *commitment* can be seen as a cognitive motivation to maintain the relationship (Arriaga and Agnew, 2001). Commitment in dating relationships must be nuanced since adolescents’ relationships are less likely to be high committing. Instead, we suggest that adolescent commitment would be best measured by indicators of how much they value their relationship and how much they are ready to invest in it in the future. Another essential aspect of relationship quality is intimacy or *complicity*, reflecting partners’ emotional bond or proximity (Li and Chan, 2012). Together, these concepts serve as a foundation for developing a dating relationship quality instrument assessing the level of emotional closeness between partners, the value attributed to the relationship, and a subjective evaluation of the relationship.

## Objectives

This study aimed to develop a new measure of dating relationship quality by assessing constructs of satisfaction (i.e., subjective evaluation), commitment (i.e., cognitive motivation), and complicity (i.e., emotional proximity). Based on previous findings, the subscales of the Relationship Quality Inventory for Adolescents (RQI-A) were hypothesized to be positively associated with problem-solving and relational skills (Furman et al., 2008; Todorov et al., 2021) and negatively linked with attachment insecurities, conflict management difficulties, and DV experiences (Mikulincer and Shaver, 2013; Orpinas et al., 2013; Todorov et al., 2021). We expected relationship-related experiences to be significantly associated with adolescents’ perception of the quality of their dating relationship. For example, we expected adolescents with greater conflict management and relational skills to report better relationship quality. Conversely, adolescents reporting the occurrence of DV, difficulties in managing conflict, and higher levels of attachment insecurities were expected to have lower scores on the RQI-A subscales.

## Methods

### Participants and procedure

A sample of French-speaking dating adolescents (currently dating or have had a partner in the past 12 months) was recruited *via* social media platforms (e.g., Facebook, Instagram). Adolescents not currently dating at the time of the study were asked to complete the questionnaire while referring to their previous relationship. The total sample comprises 645 adolescents (65.9% of girls) aged between 14 and 19 years, most of whom were of Quebec origin (81.6%). Most participants were currently involved in a dating relationship (74%), while 26% reported having had a dating partner during the past 12 months. The online questionnaire required 15 min to complete, and 25 Amazon gift cards ($25 each) were drawn among participants. In addition to the RQI-A, various relationship-related measures were included to assess the convergent validity and were selected based on their psychometric properties, brevity, and age-appropriate validity. Ethical approval was granted by the ethics committee of the Université du Québec à Montréal. The total sample was randomly divided to conduct exploratory and confirmatory factor analyses. Demographics and differences between the subsamples are presented in Table 1.

**TABLE 1:** Subsamples’ characteristics and differences.

Variables	Sample 1 (*n* = 310)	Sample 2 (*n* = 335)	*p^a^*
		
	M (SD)	M (SD)	
Age (14–19)	16.72 (1.48)	16.85 (1.45)	0.267
Girls (%)	65.8	66	0.965
Cisgender identity (%)	94.5	95.8	0.423
Heterosexual orientation (%)	68.9	72.2	0.370
Current relationship (%)	73.9	74	0.963
First relationship (%)	44.2	40.3	0.317
Length of the relationship (1–61 months)	11.99 (11.54)	10.69 (9.50)	0.120
Quebecer (%)	83.8	79.7	0.183
Living with both parents (%)	45.8	46.6	0.846
Parents’ university degree (%)	45.6	48.7	0.442

^a^*p*-values for *t*-tests or χ^2^.

### Measures

#### Relationship quality

The French version of the RQI-A was developed based on the guidelines proposed by DeVellis (2016). Following a literature review on the characteristics of healthy dating relationships, a pool of 21 items was created to assess three theoretical subscales of satisfaction, commitment, and complicity. A preliminary content validity evaluation was conducted: (1) four adolescents (15–16 years old) from a focus group evaluated the items’ clarity, (2) four caseworkers from partner organizations rated their relevance to dating relationships, and (3) four researchers from another research team assessed their level of correspondence with their theoretical construct. Following this preliminary evaluation, some items were reworded to better suit adolescents’ developmental level or their theoretical construct. For example, “I am sure that our relationship will last a long time” was changed to “I would like my relationship to last a long time,” which better reflects our definition of commitment (i.e., a cognitive motivation to engage in a stable relationship). The French version of the RQI-A was then administered to the current sample with the goal of extracting a shorter measure. Adolescents indicated how much they agreed with each statement on a 5-point Likert scale (1 = Strongly disagree to 5 = Strongly agree).

#### Relational skills

The Relational Skills Inventory for Adolescents (RSI-A) (Fortin et al., 2021) comprises 16 items answered on a 5-point Likert scale (1 = Never to 5 = Always) measuring three different relational skills (i.e., assertiveness, support, and individuality). The internal consistency of the three subscales was adequate (ω = 0.71–0.79).

#### Conflict management strategies

The Conflict Resolution Styles Inventory (CRSI) (Fortin et al., 2020) comprises 16 items answered on a 5-point Likert scale (1 = Never to 5 = Always) measuring three different conflict management strategies (i.e., conflict engagement, withdrawal, and problem-solving). The internal consistency of the three subscales was adequate (ω = 0.76–0.86).

#### Dating violence

The Conflict in Adolescent Dating Relationships Inventory-Short Form (CADRI-SF) (Fernández-González et al., 2012) measured various violent behaviors. Items were answered using a 4-point Likert scale (1 = Never to 4 = six times or more). Given the low incidence of DV, the victimization and perpetration subscale scores were dichotomized to indicate whether adolescents had experienced or perpetrated at least one act of any form of DV.

#### Romantic attachment

The Experience in Close Relationships - Short Form (ECR) (Lafontaine et al., 2016) comprises 12 items answered on a 7-point Likert scale (1 = Strongly disagree to 7 = Strongly agree) measuring two dimensions of romantic attachment (i.e., attachment anxiety and avoidance of proximity). The internal consistency of the two subscales was good (ω = 0.84 and 0.87).

## Analytic approach

Analyses were conducted using R (R Core Team, 2021) and the following packages: *Lavaan* v. 0.6-10 (Rosseel, 2012), *Psych* v. 2.1.9 (Revelle, 2020), *SemPlot* v. 1.1.2 (Epskamp, 2019), *Misty* v. 0.4.3 (Takuya, 2021), and *Tidyverse* v. 1.3.1 (Wickham et al., 2019). In the first subsample, an initial exploratory factor analysis (EFA) was conducted to examine the latent structure of the RQI-A. Oblique rotation and unweighted least squares estimation and minimum residual extraction methods were selected to allow the latent factors to correlate with one another and to account for the use of ordinal measurement scales. The Kaiser–Meyer–Olkin (KMO) index and the Bartlett sphericity test were used to verify the adequacy of the factor structure and the interitem correlations. A method agreement procedure, which compares results from multiple convergence indicators (e.g., optimal coordinates, parallel analysis, Kaiser criterion, Velicer’s MAP), was used to select the optimal factor solution. Then, the factor model extracted by the EFA was cross-validated in the second subsample using a confirmatory factor analysis (CFA) with WLSMV estimator. Several fit indices were examined to assess the correspondence between the theoretical and observed models (i.e., RMSEA, SRMR, CFI, TLI, aGFI; Hooper et al., 2008; Hair et al., 2010; Sahoo, 2019). A second-order CFA was also tested to explore whether a second-order relationship quality construct could account for the distinct but related first-order factors. The nested models were then compared using a chi-square difference test for which a non-significant value (α ≥ 0.05) indicates a non-significant decrease in model fit. Finally, to assess the convergent and divergent validity of the RQI-A, Pearson’s correlations with other relationship-related measures were examined.

## Results

Inspection of descriptive statistics indicated that 5.74% cases were incomplete with only 0.46% of missing values ranging from 0 to 1.55% across all variables. Little’s MCAR test was not significant (χ^2^ = 481.90, *p* = 0.06), therefore full information maximum likelihood (FIML) (Arbuckle, 1996; Enders, 2001) was used to estimate the missing data.

### Exploratory factor analysis

Inspection of loading coefficients revealed that six items presenting loadings smaller than 0.40 were removed from the initial scale resulting in 15 items (Table 2). The second EFA converged toward a simple 2-factor structure of Commitment (five items) and Connectedness (10 items). The Connectedness factor reflects the fusion between items of the Satisfaction and Complicity theoretical subscales. The five items with the highest loading coefficients were retained to reduce the Connectedness subscale. The method agreement procedure supported the choice of a two-factor structure. The final model explained 62% of the variance, the KMO index of 0.92 indicated excellent sampling adequacy (range 0.90–0.96), and the Bartlett sphericity test was significant (χ^2^ = 1979.05, *p* < 0.001). The distribution of residuals and the residual correlation coefficients matrix were examined; the residuals were normally distributed, and only 0.07% were greater than 0.05, thus indicating a good fit (Yong and Pearce, 2013). Internal consistency of the RQI-A subscales was excellent, with omega coefficients of 0.89 (Connectedness) and 0.89 (Commitment).

**TABLE 2:** Means, standard deviations, and factor loadings of the exploratory factor analysis (EFA) (*n_1_* = 310).

Items	Loadings					
	21-item		15-item		10-item	
	F1	F2	F1	F2	F1	F2
13. My relationship matches my expectations	0.923		0.912		0.840	
10. I am satisfied with my relationship	0.774		0.790		0.796	
7. I am happy in my relationship	0.744		0.741		0.742	
5. In general, we get along well no matter the situation	0.650		0.743		0.733	
8. We understand each other without having to tell each other everything	0.588		0.671		0.657	
1. I sometimes have doubts about my relationship (R)	0.702		0.669		−	−
16. I find that my partner makes me a better person	0.600		0.648		−	−
19. My relationship allows me to grow	0.605		0.610		−	−
4. I wonder if I would be happier with someone else (R)	0.702		0.544		−	−
18. My relationship requires more effort than I am willing to give (R)	0.498		0.456		−	−
3. My relationship is very important to me		0.936		0.880		0.902
6. I would like my relationship to last a long time		0.687		0.775		0.786
9. I am willing to make efforts to preserve my relationship		0.692		0.771		0.782
12. I invest in my relationship despite the difficulties encountered		0.630		0.601		0.614
15. My relationship isn’t always perfect, but it’s worth it		0.442		0.455		0.464
2. We are very close to each other			−	−	−	−
11. We have fun together, even when we have nothing special to do or say			−	−	−	−
14. We have very little in common (R)			−	−	−	−
17. We often confide in each other			−	−	−	−
20. We have our own inside jokes that only we can understand			−	−	−	−
21. I don’t feel ready to fully commit to my relationship (R)			−	−	−	−
Eigenvalue	5.35	3.21	4.90	2.80	3.22	2.95
Variance accounted (%)	26	15	33	19	32	30
M (SD)	3.84 (0.80)	4.44 (0.67)	3.84 (0.80)	4.44 (0.67)	4.03 (0.78)	4.44 (0.67)

F1 = Connectedness, F2 = Commitment; Coefficients greater than 0.40 are presented. (R) means that the items were reverse coded.

### Confirmatory factor analysis

Fit indices for the 15- and 10-item models were compared (Table 3). Results suggested a better fit of the 10-item vs. the 15-item model compared to expected values: RMSEA ≤ 0.06, SRMSR ≤ 0.08, CFI ≥ 0.95, and TLI ≥ 0.95, aGFI ≥ 0.95 (Hooper et al., 2008; Sahoo, 2019). As such, the more parsimonious 10-item model was retained. Internal consistency of the Connectedness and Commitment subscales was excellent (ω = 0.90 and 0.87). Finally, a second-order model was compared to the first-order model (Table 3). The result of the chi-square difference test was non-significant [χ^2^(1) = 100.04, *p* = 1], indicating that connectedness and commitment could be considered as a higher-order relationship quality latent construct. The final version of the RQI-A comprises ten items, which can be averaged to produce either two commitment and connectedness scores or an overall relationship quality score (the final version of the instrument and the scoring procedure can be found in the Supplementary material).

**TABLE 3:** Psychometric properties of the Relationship Quality Inventory for Adolescents (RQI-A) (*n_2_* = 335).

Model	TLI	RMSEA [90% CI]	SRMR	CFI	aGFI
15 items	0.93	0.05 [0.03–0.06]	0.04	0.94	0.99
10 items	0.96	0.06 [0.05–0.08]	0.04	0.97	0.99
2nd-order	0.96	0.06 [0.05–0.08]	0.04	0.97	0.99

TLI, Tucker Lewis index, RMSEA, root mean square error of approximation; SRMR, standardized root mean square residual; CFI, comparative fit index; aGFI, adjusted goodness-of-fit.

### Convergent and divergent validity

Convergent and divergent validity for the RQI-A were assessed within the total sample (Table 4). Connectedness converged with relational (assertiveness, support, individuality) and problem-solving skills, and diverged from destructive conflict management strategies (conflict engagement, withdrawal), attachment insecurities (anxiety and avoidance), and DV victimization and perpetration. Commitment was positively associated with assertiveness, support, and problem-solving, and was negatively associated with withdrawal and attachment avoidance. No associations were found with individuality, conflict engagement, anxiety, DV victimization, and perpetration.

**TABLE 4:** Convergent and divergent validity of the Relationship Quality Inventory for Adolescents (RQI-A) (*n* = 645).

Variables	Pearson’s correlations	
	Connectedness	Commitment
**Relationship skills**		
Assertiveness	0.498**	0.400**
Support	0.476**	0.529**
Individuality	0.190**	0.023
**Conflict management**		
Conflict engagement	−0.188**	–0.045
Withdrawal	−0.427**	−0.322**
Problem-solving	0.441**	0.396**
**Romantic attachment**		
Anxiety	−0.229**	0.021
Avoidance	−0.612**	−0.601**
**Dating violence**		
Perpetration	−0.097*	0.033
Victimization	−0.189**	–0.028

*p < 0.05, **p < 0.01.

## Discussion

This study aimed to validate a new measure of adolescent dating relationship quality, initially developed to assess three theoretical constructs of Satisfaction, Complicity, and Commitment. Instead, the current findings supported a two-factor structure of Connectedness and Commitment as reflected by an excellent fit of the observed model. Items from the Complicity and Satisfaction dimensions fused to form one unique factor of Connectedness. This new subscale includes items such as “My relationship matches my expectations” and “We understand each other without having to tell each other everything,” suggesting that relationship satisfaction in adolescence is closely linked to feelings of emotional proximity and getting along with one another. Interestingly, none of the negatively worded items (e.g., I wonder if I would be happier with someone else) were retained in the final 10-item model. Including negative items is often recommended to reduce acquiescence and extreme response bias. However, it can also increase the risk of misinterpretation and mistakes when answering the questions (Sauro and Lewis, 2011; Cole et al., 2019; Pastor et al., 2020), which could explain why negative items had lower loadings and were not retained in the final version of the RQI-A.

Results indicated that the internal consistency was excellent, with omega coefficients ranging from 0.87 to 0.90 in both samples. As expected, subscales of the RQI-A demonstrated convergence with indicators of healthy relationship behaviors (relational skills and problem-solving) and divergence with indicators of destructive relationship behaviors and difficulties (negative conflict management strategies, attachment insecurities, and DV). In line with previous research (Woodin, 2011; Li and Chan, 2012; Spencer et al., 2022), small effect sizes were observed for DV, conflict engagement, individuality, and attachment anxiety, whereas moderate effects were found for support, withdrawal, and problem-solving, and large effect were noted for assertiveness and attachment avoidance. However, Commitment did not correlate with DV, conflict engagement, attachment anxiety, and individuality. Even if adolescents are experiencing difficulties with their partner, they still value and want to pursue their relationship. This finding reflects the salience of early dating experiences in adolescence (Furman, 2018) and may explain why adolescents’ levels of commitment did not vary according to their relationship difficulties.

### Implications and applications

Dating relationships involve many relational challenges and opportunities for growth. These experiences are increasingly important to adolescents’ development and wellbeing as they provide a source of support and affection while also allowing them to further develop their communication and conflict management skills (Simon and Furman, 2010; Gómez-López et al., 2019). Given that many DV prevention programs now focus on promoting the formation of healthy dating relationships (Exner-Cortens et al., 2019; Niolon et al., 2019; Miller et al., 2020), having access to a reliable and valid measure of dating relationship quality will help researchers assessing the efficacy of these programs on relational outcomes.

Our results suggest that adolescent commitment to their relationship does not depend on the destructive behaviors they use or the relational difficulties they experience with their partner. This finding is in line with prior work suggesting that even among couples reporting the presence of DV, adolescents report high levels of care, love, and self-disclosure (Giordano et al., 2010). Previous studies also found that victimization experiences did not predict relationship dissolution among adolescents (Soller et al., 2020; Muñoz-Rivas et al., 2021). The current findings showed that these difficulties are negatively associated with connectedness and suggest that adolescents may opt to remain involved in an unsatisfying relationship to meet social standards and expectations. This is consistent with Muñoz-Rivas et al. (2021), who reported that the vast majority (79.8%) of adolescent victims of DV intended to stay in their abusive relationship. Therefore, gaining a better understanding of dating dynamics by examining the predictors and correlates of adolescent relationship quality could yield relevant information that can be used to inform and enhance existing prevention initiatives.

### Limitations and future directions

Although this study provides evidence that the RQI-A is a reliable and valid measure of dating relationship quality, some limitations must be considered. The instrument was validated among a French-speaking sample of adolescents; it would be helpful to validate the RQI-A in different languages for various youth samples. Since we had to divide our sample to conduct EFA and CFA, larger samples should be used in future studies to further validate our findings and have sufficient power to test for gender invariance of the instrument. It would also be interesting to test for invariance between adolescents currently dating and those whose relationship has ended. Finally, using a cross-sectional design limited our ability to draw conclusions regarding the stability of the RQI-A scores, an essential dimension of reliability. Therefore, future studies should conduct a test-retest procedure and replicate our findings in more diverse samples.

## Conclusion

In sum, the current findings provide support for the psychometric soundness of the RQI-A. Researchers now have access to a measure of adolescent dating relationship quality with documented validity and reliability. Using the RQI-A in future studies could help better understand the predictors and correlates of adolescent dating relationship quality.

## Data availability statement

The raw data supporting the conclusions of this article will be made available by the authors, without undue reservation.

## Ethics statement

The studies involving human participants were reviewed and approved by Comité Institutionnel d’Éthique de la Recherche Avec des Êtres Humains–Université du Québec à Montréal. Written informed consent from the participants’ legal guardian/next of kin was not required to participate in this study in accordance with the national legislation and the institutional requirements.

## Author contributions

AF conceived the study, participated in its design and coordination, performed the statistical analyses, led the interpretation of the data, and drafted the manuscript. LF assisted in the interpretation of the data and the writing of the manuscript. AP assisted in the interpretation of the data and the editing of the manuscript. MH led the conception and design of the study, contributed to the interpretation of the data, and assisted in the editing of the manuscript. All authors read and approved the final manuscript.
