# Supplementary material for: Relationship quality among dating adolescents: Development and validation of the Relationship Quality Inventory for Adolescents
Source: Front Psychol. 2022 Oct 14;13:1026507. doi: 10.3389/fpsyg.2022.1026507 (PMC9614222; doi:10.3389/fpsyg.2022.1026507)
Supplement: Supplementary file 1 [file Data_Sheet_1.pdf]

Supplementary Material

INVENTAIRE DE LA QUALITÉ DES RELATIONS AMOUREUSES À L'ADOLESCENCE

[*RELATIONSHIP QUALITY INVENTORY FOR ADOLESCENTS*]

Indique à quel point tu es d'accord avec chacun des énoncés suivants.
[*Indicate how much you agree with each of the following statements*.]

**Fortement en désaccord** *[Strongly disagree]*	**En désaccord** *[Disagree]*	**Ni en accord ni en désaccord** *[Neither agree nor disagree]*	**En accord** *[Agree]*	**Fortement en accord** *[Strongly agree]*
**1**	**2**	**3**	**4**	**5**

	**1**	**2**	**3**	**4**	**5**
1. Ma relation est vraiment importante pour moi. [*My relationship is very important to me*]	○	○	○	○	○
2. En général, nous nous entendons bien, peu importe la situation. [*In general, we get along well no matter the situation*]	○	○	○	○	○
3. J'aimerais que ma relation dure longtemps. [*I would like my relationship to last a long time*]	○	○	○	○	○
4. Je suis heureux·se dans ma relation. [*I am happy in my relationship*]	○	○	○	○	○
5. Je suis prêt·e à faire des efforts pour préserver ma relation. [*I am willing to make efforts to preserve my relationship*]	○	○	○	○	○
6. Nous nous comprenons, sans avoir besoin de tout se dire. [*We understand each other without having to tell each other everything*]	○	○	○	○	○
7. Je m'investis dans ma relation malgré les difficultés rencontrées. [*I invest in my relationship despite the difficulties encountered*]	○	○	○	○	○
8. Je suis satisfait·e de ma relation. [*I am satisfied with my relationship*]	○	○	○	○	○
9. Ma relation n'est pas toujours parfaite, mais elle en vaut la peine. [*My relationship isn't always perfect, but it's worth it*]	○	○	○	○	○
10. Ma relation correspond à mes attentes. [*My relationship matches my expectations*]	○	○	○	○	○

SCORING PROCEDURE

Subscales are computed by averaging the score of their respective items.
Higher scores reflect greater connectedness or commitment.

Connexion [Connectedness]

2. En général, nous nous entendons bien, peu importe la situation.
[*In general, we get along well no matter the situation*]

4. Je suis heureux·se dans ma relation.
[*I am happy in my relationship*]

6. Nous nous comprenons, sans avoir besoin de tout se dire.
[*We understand each other without having to tell each other everything*]

8. Je suis satisfait·e de ma relation.
[*I am satisfied with my relationship*]

10. Ma relation correspond à mes attentes.
[*My relationship matches my expectations*]

Engagement [Commitment]

1. Ma relation est vraiment importante pour moi.
[*My relationship is very important to me*]

3. J'aimerais que ma relation dure longtemps.
[*I would like my relationship to last a long time*]

5. Je suis prêt·e à faire des efforts pour préserver ma relation.
[*I am willing to make efforts to preserve my relationship*]

7. Je m'investis dans ma relation malgré les difficultés rencontrées.
[*I invest in my relationship despite the difficulties encountered*]

9. Ma relation n'est pas toujours parfaite, mais elle en vaut la peine.
[*My relationship isn't always perfect, but it's worth it*]
